# Egg-laying by female *Aedes aegypti* shapes the bacterial communities of breeding sites

**DOI:** 10.1186/s12915-023-01605-2

**Published:** 2023-04-26

**Authors:** Katherine D. Mosquera, Luis Eduardo Martínez Villegas, Gabriel Rocha Fernandes, Mariana Rocha David, Rafael Maciel-de-Freitas, Luciano A. Moreira, Marcelo G. Lorenzo

**Affiliations:** 1 Vector Behavior and Pathogen Interaction Group, René Rachou Institute-FIOCRUZ, Belo Horizonte, Minas Gerais Brazil; 2 Department of Entomology, The Ohio State University, 2001 Fyffe Rd., Room 232 Howlett Hall, Columbus, OH 43210 USA; 3 Mosquito Vectors: Endosymbionts and Pathogen-Vector Interactions Group, René Rachou Institute-FIOCRUZ, Belo Horizonte, Minas Gerais Brazil; 4 Biosystems Informatics, René Rachou Institute-FIOCRUZ, Belo Horizonte, Minas Gerais Brazil; 5 Laboratory of Hematozoa Transmitting Mosquitoes, Oswaldo Cruz Institute-FIOCRUZ, Rio de Janeiro, Brazil

**Keywords:** *Aedes aegypti*, Microbiota, Breeding sites, Oviposition, Niche construction

## Abstract

**Background:**

*Aedes aegypti*, the main arboviral mosquito vector, is attracted to human dwellings and makes use of human-generated breeding sites. Past research has shown that bacterial communities associated with such sites undergo compositional shifts as larvae develop and that exposure to different bacteria during larval stages can have an impact on mosquito development and life-history traits. Based on these facts, we hypothesized that female *Ae. aegypti* shape the bacteria communities of breeding sites during oviposition as a form of niche construction to favor offspring fitness.

**Results:**

To test this hypothesis, we first verified that gravid females can act as mechanical vectors of bacteria. We then elaborated an experimental scheme to test the impact of oviposition on breeding site microbiota. Five different groups of experimental breeding sites were set up with a sterile aqueous solution of larval food, and subsequently exposed to (1) the environment alone, (2) surface-sterilized eggs, (3) unsterilized eggs, (4) a non-egg laying female, or (5) oviposition by a gravid female. The microbiota of these differently treated sites was assessed by amplicon-oriented DNA sequencing once the larvae from the sites with eggs had completed development and formed pupae. Microbial ecology analyses revealed significant differences between the five treatments in terms of diversity. In particular, between-treatment shifts in abundance profiles were detected, showing that females induce a significant decrease in microbial alpha diversity through oviposition. In addition, indicator species analysis pinpointed bacterial taxa with significant predicting values and fidelity coefficients for the samples in which single females laid eggs. Furthermore, we provide evidence regarding how one of these indicator taxa, *Elizabethkingia*, exerts a positive effect on the development and fitness of mosquito larvae.

**Conclusions:**

Ovipositing females impact the composition of the microbial community associated with a breeding site, promoting certain bacterial taxa over those prevailing in the environment. Among these bacteria, we found known mosquito symbionts and showed that they can improve offspring fitness if present in the water where eggs are laid. We deem this oviposition-mediated bacterial community shaping as a form of niche construction initiated by the gravid female.

**Supplementary Information:**

The online version contains supplementary material available at 10.1186/s12915-023-01605-2.

## Background

The mosquito *Aedes aegypti* (Linnaeus, 1762) is the main vector of the arboviruses causing dengue, yellow fever, Zika, and chikungunya. Its wide distribution across tropical and subtropical regions in close association with urban areas makes this mosquito a major threat to human health [1, 2]. Urban houses represent suitable mosquito habitats with a reduced number of predators, diverse sugar sources, widely available blood sources (as well as resting places for gravid females), and a variety of water-holding containers accessible for egg-laying and larval development [3, 4]. In these environments, artificial containers that accumulate water (e.g., flower pots, discarded plastic or metallic cups, and tires act as breeding sites [3, 5]. In most cases, these containers collect rainwater, which is a poor source of nutrients.

Biotic and abiotic elements present in water are known to drive the selection of oviposition sites by gravid females. These include the presence of conspecifics and/or predators, organic matter, surrounding vegetation, color, moisture, salinity, ammonium, and phosphate [6–9]. Furthermore, microbial communities have been shown to influence *Ae. aegypti* oviposition choices [10–12]. Indeed, females locate suitable breeding sites using microbe-emitted infochemicals [13]. Oviposition choices probably endure selection pressures because microorganisms serving as larval food can also establish intricate host-bacterial community networks, eventually defining symbiotic relations [11].

The origin of the microbial communities that colonize mosquitoes and the relative contribution of the environment to their acquisition are still debated [14, 15]. It has been shown that part of mosquito-associated bacteria is acquired during early life stages in larval habitats [16–19]. Besides, the bacterial communities present in *Ae. aegypti* larvae are influenced by the aquatic environment where they develop [9, 16]. Furthermore, some members of the bacterial community can be transstadially transmitted to adults [16, 18, 20–22].

Mosquito females can add key microbial associates during egg-laying, affecting the microbial community within the breeding site [17]. This may promote symbiont dispersal, providing offspring with specific microbial inocula rather than leaving their acquisition to chance [23]. Bacteria recovered from immature stages and adults have already been detected on egg surfaces [16, 22]. Indeed, mosquitoes can transfer bacteria to their oviposition sites and pick them up from the water they emerged from [20, 22]. It has been suggested that transmission of maternal microbiota to larval breeding sites could occur directly, through egg smearing or transovarial transmission; or indirectly during egg-laying when females might unintentionally inoculate microbes into oviposition sites [24].

Although the properties of the external environment influence the bacterial communities of a niche, the dissemination of microbial cells from eukaryotic hosts can also impact the composition and traits of the microbiota in the immediate environment [23, 25]. Considering that *Ae. aegypti* exploits small and temporary water containers, altering the bacterial community of larval habitats could have a substantial impact on larval fitness [21]. If verified, this ability could elucidate its ability to exploit confined nutrient-scarce habitats. Environment-modifying capacities exerted by parental individuals and their offspring during ontogenesis is a tenet of niche construction theory [26, 27]. Within this conceptual framework, the phenomenon of developmental niche construction can occur via chemical excretion, generation of physical structures (e.g., beaver dams), or due to the physiological properties of symbionts [28, 29]. Whether mosquito larval habitats represent a case of niche construction is still unknown.

Our study evaluated whether gravid female mosquitoes shape the bacterial community of the breeding site as a strategy to enhance offspring fitness. To address our hypothesis, we investigated whether gravid females (i) act as mechanical vectors of bacteria, (ii) modulate the bacterial community in water-holding containers through oviposition, and (iii) promote bacteria (acting as oviposition indicators) that enhance progeny fitness.

## Methods

### Mosquito rearing

*Aedes aegypti* (F2) were obtained from a Brazilian laboratory colony (BR URCA) established from eggs collected in ovitraps in the Urca district of Rio de Janeiro city. All mosquitoes used in the experiments were maintained under insectary conditions at 28 ± 2 °C, 70 ± 10% relative humidity, and a 12:12 light/dark photoperiod. Larvae were reared in plastic trays containing non-chlorinated water and fed half a tablet of TetraMin fish food (Tetra) every day. Pupae were transferred from rearing trays to cardboard cages in plastic flasks, after which adults emerged. Adults were offered 10% sucrose solution ad libitum. Females were blood-fed 7 days post-emergence on a Hemotek Membrane Feeding System (Hemotek Ltd) using human blood. Human blood used to feed adult mosquitoes was obtained from a blood bank (Fundação Hemominas, Belo Horizonte, Minas Gerais, Brazil), according to the terms of an agreement with Instituto René Rachou, Fiocruz Minas (OF.GPO/CCO agreement-Nr 224/16). Pilot experiments revealed that this mosquito population has its oviposition peak 72 h after a blood meal. Only fully engorged females were collected for further assays.

### Mechanical transmission of bacteria

#### Experimental design

To assess whether *Ae. aegypti* females can mechanically transfer viable bacteria to solid culture media, a single female was released in a cardboard cage (brand new, cleaned with 70% ethanol-soaked paper wipes, and exposed to 15 min of UV light in a biosafety cabinet) presenting a Petri dish at the bottom loaded with either LB or blood agar media. Five replicates were performed per culture medium tested, plus two environmental control plates per medium type.

After 24 h, females were removed from the cages, pooled, and washed with 1 ml of sterile phosphate-buffered saline (PBS) for 10 min. Moreover, a swab of the wall and bottom of the cardboard cage was collected and placed in PBS (1 ml) for 10 min. Subsequently, an aliquot (50 µl) of these PBS washes, both from the body surfaces and the cage swab, was inoculated on LB (Lysogeny Broth) and blood agar plates, separately. The plates were incubated for up to 48 h. Negative control plates with only sterile PBS resulted in no colonies.

#### DNA extraction and PCR amplification

Bacterial isolates were examined and characterized according to their features. Colonies with visually distinct morphologies were isolated from each medium, followed by total genomic DNA extraction using the DNeasy Blood & Tissue Kit (Qiagen), according to the manufacturer’s manual. A reagent blank extraction was performed as a negative control of the process.

The full length of the bacterial 16S ribosomal RNA (16S rRNA) gene (~ 1500pb) was amplified by the pair of primers 27F (5′-AGAGTTTGATCMTGGCTCAG-3′) and 1492R (5′-TACGGYTACCTTGTTACGACTT-3′). Polymerase chain reactions (PCR) were carried out in a 25 µL final volume using 0.50 µl of 5U/µl GoTaq Polymerase (Promega), 1.50 µl of 25 mM MgCl2, 0.50 µl of 10 mM dNTP mixture, 5 µl of 5X reaction buffer, 10 µM of each primer and 2.5 µl of template DNA. Amplification consisted of an initial denaturation at 95 °C for 2 min, 30 cycles of 95 °C for 30 s, 55 °C for 30 s, and 72 °C for 1 min 40 s, followed by a final extension at 72 °C for 5 min. A PCR amplification control was performed. Reactions and negative controls were analyzed by electrophoresis in a 1% agarose gel. Controls, both from DNA extraction and PCR, showed no amplified bands.

#### Sanger sequencing and taxonomic identification

PCR products were purified using the ReliaPrep DNA Clean-up and Concentration System (Promega) following the manufacturer’s protocol. Sequencing reactions were conducted using the BigDye Terminator v3.1 Cycle Sequencing Kit (Thermo Fisher Scientific). Three primers (two forward and one reverse) were used to generate amplicons for Sanger sequencing [27F, 515F (5′-GTGCCAGCMGCCGCGGTAA-3′), and 1492R]. The combination of sequence data obtained with these three amplicons generates a contiguous sequence that encompasses most of the full 16S rRNA gene [30]. Sequencing was performed on an ABI 3730 DNA sequencer.

The sequenced reads were assembled using the software Geneious Prime v2019.0.4. Bacterial taxonomic classification was performed using the SILVA Alignment, Classification and Tree Service with a minimal identity with query sequences of 95%.

### Changes in the bacterial profile of breeding sites

#### Experimental design

**Fig. 1:**
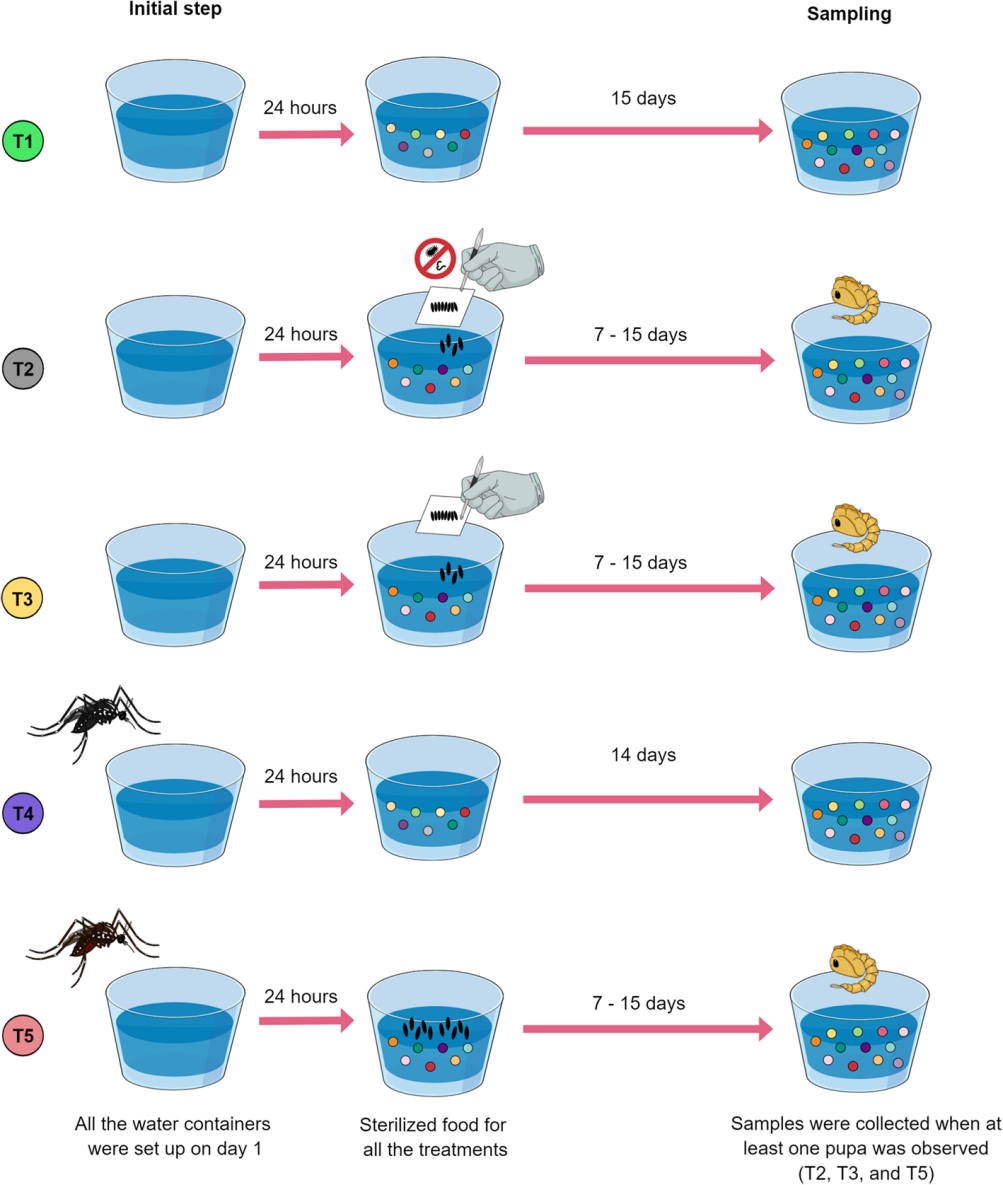
Detection of profile shifts on microbial communities induced by oviposition-related inputs. An experimental scheme was elaborated to dissect the effects of the act of oviposition from other sources affecting microbial profiles observed after mosquito-water interactions. DNA extracted from water samples belonging to five different treatments was subjected to amplicon-oriented sequencing to characterize the structure of their bacterial communities. Treatment 1 (T1): environmental control (type I water plus sterilized food). Treatment 2 (T2): manually-deposited sterilized mosquito eggs. Treatment 3 (T3): manually-deposited non-sterilized eggs. Treatment 4 (T4): single sugar-fed females interacting with container water. Treatment 5 (T5): single gravid female (72 h post-blood-feeding) allowed to lay eggs

To test whether *Ae. aegypti* females modify breeding site community composition through oviposition, an experiment with five different treatments was designed (Fig. 1). Ten replicates per treatment were carried out using cardboard cages presenting a plastic cup with 80 ml of type I water and 500 µl of sterilized food. This diet was prepared by dissolving finely groundfish food in type I water and autoclaving it for 20 min at 120 °C. All water containers were set up on day one with sterilized food added on day 2.

Treatment 1 (T1) acted as an environmental control (type I water plus sterilized food). Treatment 2 (T2) was developed using sterilized mosquito eggs that were manually deposited. Eggs were sterilized using 70% ethanol for 5 min, followed by a wash in a 3% bleach and 0.1% benzalkonium chloride (Quatermon 30, Chemitec, Brazil) solution for 3 min, an additional wash in 70% ethanol for 5 min, and rinsing three times in sterile water. The sterile condition of eggs was confirmed by negative PCR amplification of the 16S rRNA gene V4 hypervariable region using the primers 515F and 806R (5′-GGACTACHVGGGTWTCTAAT-3′). Besides, this was reinforced by the absence of bacterial growth from sterilized eggs transferred to LB broth, which indicated no viable bacteria were present. Treatment 3 (T3) was developed using manually deposited non-sterilized eggs. Eggs, both for T2 and T3, were derived from groups of gravid females that oviposited on pieces of filter paper, which were stored under insectary conditions until needed (but not longer than one month). Treatment 4 (T4) was developed with a sugar-fed female that was held for 24 h without access to drinking to assure that it would interact with the water in the container and thus ensure physical contact control for mosquitoes that cannot lay eggs. Treatment 5 (T5) was developed with a gravid female (72 h post-blood-feeding) that was allowed to lay eggs. For both, T4 and T5, females were removed from cardboard cages after a 24 h exposure interval. Once females were removed, the number of eggs laid in each T5 replicate was counted using a magnifying glass (Additional file 1). This allowed us to calculate an average number subsequently used for manually depositing eggs (51 eggs) in T2 and T3 replicates.

For T2, T3, and T5, water samples were collected when at least one pupa was detected. For T4 and T1, water samples were collected on days 15 and 16, respectively. As pupation represents a developmental checkpoint in the holometabolous cycle [31], we considered this criterion as the basis for the sampling as it represents an environment that has successfully sustained the development of larvae.

#### DNA extraction and high-throughput sequencing

Each water sample was aseptically filtered through a polyethersulfone membrane (0.22 μm pore size, 50 mm diameter) using vacuum-driven filters (Biofil) and a vacuum-pressure pump (Millipore). Filter membranes were cut into small pieces using a stainless steel scalpel and placed in sterile tubes. A control using ultrapure water was carried out to verify whether the membrane or filtration process could introduce any contamination. Bacterial genomic DNA was extracted from bacterial cells retained on each filter membrane using the DNeasy PowerSoil Kit (Qiagen), following the manufacturer’s methods. A reagent blank extraction was the control of the DNA extraction process. DNA sample concentration was measured using a Qubit fluorescence assay (Invitrogen). All DNA samples were concentrated in a vacufuge concentrator (Eppendorf) and sent for amplicon sequencing (16S rRNA, V4 region primers) on an Illumina HiSeq PE250 instrument at Novogene Bioinformatics Technology Co., Ltd. (Beijing, China). Since the controls, both from the filtration process and the DNA extraction resulted in negative PCR amplification, they were not further processed and were not sequenced (Additional file 2).

#### Bioinformatics analysis and taxonomic assignment

Raw sequence data generated were processed using the DADA2 pipeline v1.6.0 [32] to identify Amplicon Sequence Variants (ASVs). The raw reads were trimmed to remove the primers. The forward reads were trimmed at position 180 and the reverse reads at nucleotide 150. After trimming, the reads with a maximum of 2 expected errors for the error model prediction and merging were conserved.

Taxonomic classification was assigned by TAGME [33] using Silva 138 database and the pre-built model for the amplified region. *HTSFilter* package v1.38.0 [34] was used to remove ASVs containing reads less than a cutoff value defined by calculating a Jaccard index. All the above-mentioned bioinformatics tools, plus diversity and statistical analyses downstream were executed in Rstudio v1.1.423.

#### Diversity and statistical analyses

ASVs diversity within and between samples was compared. The Simpson index (1-D) was used to measure alpha diversity. Alpha diversity metrics between groups were compared using the Kruskal–Wallis test, followed by post hoc Dunn’s multiple comparisons tests. *P*-values were adjusted using the Benjamini–Hochberg method.

A Jensen-Shannon distance matrix was used for beta diversity analysis. A principal coordinates analysis (PCoA) was conducted to visualize and interpret the overall dissimilarity in the microbial community structure among the treatments. A Permutational Multivariate Analysis of Variance (PERMANOVA) [35] was performed to explore the significance of the presence of eggs and/or the female interaction with water, on the bacterial signatures associated with each group. Additionally, a pairwise PERMANOVA [36] based on the ASV abundance matrix transformed using the Hellinger method was applied to evaluate the significance of the variance between each treatment.

The differentially abundant ASVs were detected using *DESeq2* v1.38.1 [37]. In a multivariate model, the likelihood ratio test was used to identify differentially abundant variants. For univariate analysis, the variants differing in each variable — female interaction with water and eggs presence — were identified using the Wald test. All ASVs with adjusted *P*-value < 0.01 were considered differentially abundant and were used for model construction.

A general Random Forest (RF) model was built using all the previously identified ASVs and the Gini importance of each ASV was calculated. The 30 most important ASVs were used to construct models for each variable — female interaction with water and egg presence. One thousand bootstrap analyses were performed by randomly selecting 50% of samples from the analyzed variable, building 100 trees, and calculating the importance of each ASV. The 10 most important ASVs among the 1000 tests were chosen to build a final predictive model. The model construction and performance analysis were executed using *caret* package v6.0–86 [38].

As the predictive model tested by the RF approach identified features capable of discriminating the communities based on the key experimental variables, we deemed it relevant to search for indicator taxa. This ecological analysis was executed to identify ASVs that reflect the effects that biotic and/or abiotic factors, encompassed within each treatment, exert, thus shaping the community composition. In particular, we aimed to identify ASVs whose occurrence and abundance provide evidence of the impact that oviposition and larval development (T5) had upon the breeding site bacterial consortium. The analysis was performed using the *indicspecies* package v1.7.7 [39].

### Effects of *Elizabethkingia* on larval development, mortality, and adult size

#### Selection of bacteria for larval fitness experiments

To assess if ASVs identified as an indicator of oviposition activity may have an impact on *Ae. aegypti* development, a bacterial strain belonging to the genus *Elizabethkingia* was selected for fitness experiments. As a control, we also tested *Asaia*, a bacterial symbiont widely present in the microbiota of several mosquito species [40] that has been previously shown to shorten the larval development time of *Anopheles* mosquitoes [41, 42].

*Asaia* sp. strain AE06 (GenBank accession: KR703670) was recovered from the midgut of adult females of the Paea laboratory strain, which was established in 1994 [43]. *Elizabethkingia* sp. strain VV01(GenBank accession: KU096882) was isolated from field-collected mosquitoes. Wild *Ae. aegypti* were collected in Colônia Z-10 (22°49′23.50″S; 43°10′42.93″W), a fishermen's community in Rio de Janeiro. Larvae, water, and deposited sediment were collected from two natural breeding sites, brought to the insectary, and conditioned in clean disposable cups at 27 ± 2 °C. No additional food or water was added to the cups until adult emergence. Adults were fed ad libitum with sterilized cotton soaked in sterilized 10% sucrose solution until midgut dissection. Ice-anesthetized adult female mosquitoes were surface-sterilized in 70% ethanol for 1 min and rinsed in sterile PBS. As surface sterilization control, individuals were rinsed in sterile PBS, which was plated on LB plates. Midguts were removed over a sterile glass slide and macerated in sterile PBS. Each midgut sample was tenfold diluted and plated on LB and tryptone soy agar plates. For the next 72 h, bacteria were screened based on colony morphology. Samples from each different bacterial morphotype were preserved and stored at − 70 °C. Bacterial DNA was extracted by a conventional boiling and freezing step. A 16S rRNA gene segment between the V1-V3 hypervariable regions was amplified by PCR using the primers 27F (5′-AGAGTTTGATCCTGGCTCAG-3′) and 536R (5′-GTATTACCGCGGCTGCTG-3′) and Sanger sequenced for taxonomic identification.

#### Experimental design

At 24 h post-hatching, 36 L1 (larval stage 1) larvae (Paea strain) per group were individually placed in the wells of three 12-well cell culture plates. Each well received 4 ml of non-chlorinated water, 3 mg of TetraMin fish food, and 100 μl of *Asaia* or *Elizabethkingia* culture suspended in PBS (OD600 = 1). Controls received 100 μl of PBS. Larval development was monitored three times a day (8:00, 12:00, and 17:00) to record mortality and molt for each insect. Developmental time was monitored up to the day all immatures reached the adult stage or died. The wing length was measured, excluding the fringe, as a proxy for adult body size [44]. During experiments, specimens were maintained at 27 ± 2 °C and 70 ± 10% relative humidity. Larvae were not antibiotic-treated before bacteria exposure. To verify *Asaia* and *Elizabethkingia* colonization in the larval guts, six L4 midguts from each group were dissected, homogenized in PBS, and plated on LB plates and an *Asaia*-specific isolation medium [45]. Isolated bacterial strains were taxonomically identified using the 16S rRNA gene sequencing procedure previously mentioned.

#### Statistical analysis

The non-parametric Kaplan–Meier survival analysis was performed to assess whether exposure to *Asaia* or *Elizabethkingia* affected the duration of total immature development time (L1 to adult), larval instars (L1, L2, L3, and L4) and the pupal stage. The effect of bacteria exposure was estimated as Hazard Ratios (HR) via Cox Proportional-Hazard models [46] considering the total immature development time, duration of each larval instar and pupae stage, and also larval survival as dependent variables. Wing lengths were compared using the Kruskal–Wallis test. Statistical analyses were carried out using R v3.2.3.

## Results

### *Aedes aegypti* females transmit bacteria mechanically

**Fig. 2:**
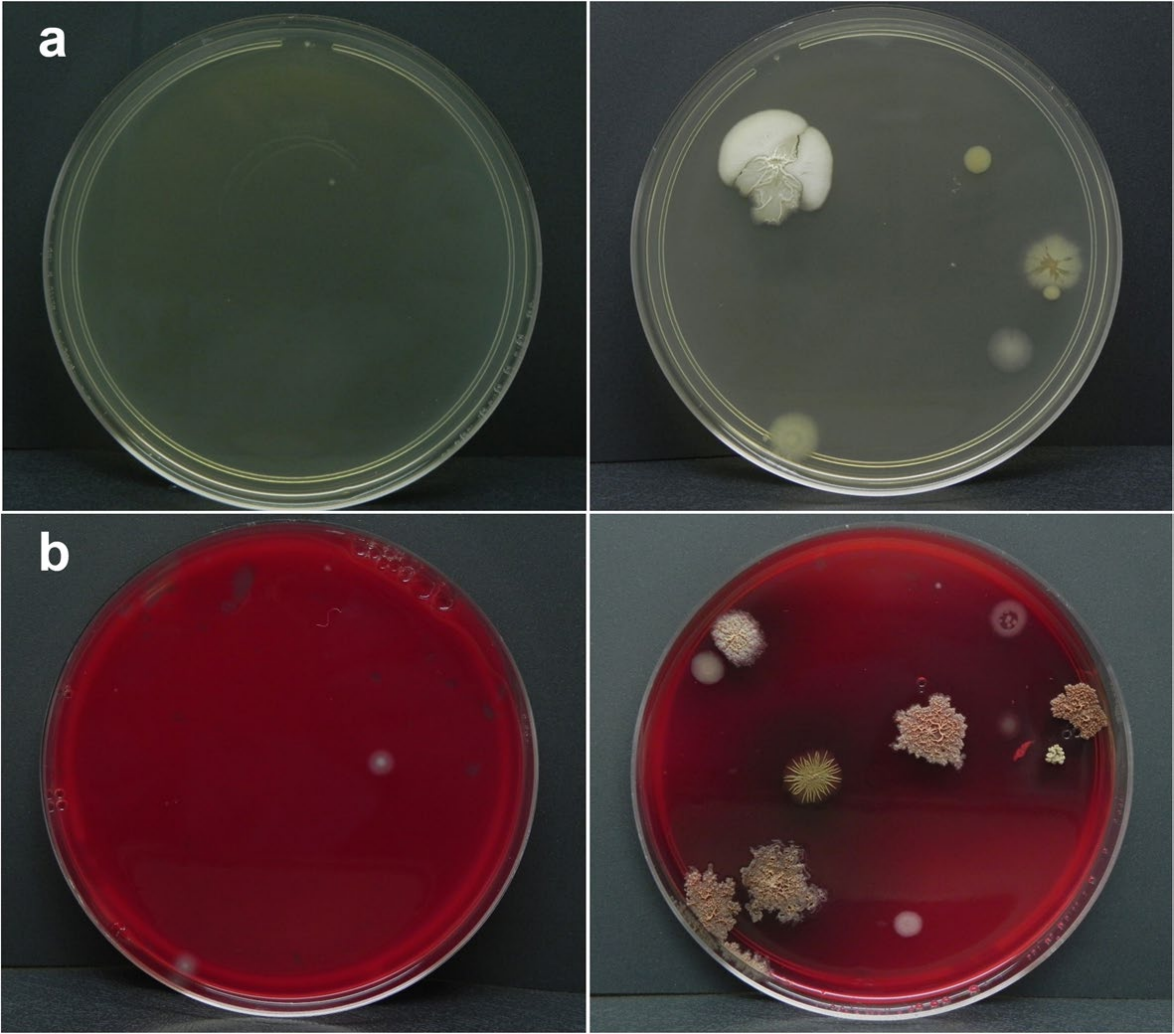
*Aedes aegypti* females transfer viable and cultivable bacteria to solid culture media. Growth of bacterial colonies on LB (**a**) and blood agar (**b**). Left, environmental control plates; right, plates exposed to interaction with a gravid female

Our results demonstrated that *Ae. aegypti* females transfer culturable viable bacteria to solid culture media (Fig. 2). To further dissect the possible sources of these bacteria, a cage swab, mosquito body washes, and environmental controls were performed for each medium tested.

A total of 28 isolates were recovered from LB plates (Supplementary Table 1, Additional file 3). Altogether, these isolates belonged to three phyla, six families, and seven genera. The bacterial diversity observed in LB plates exposed to interaction with a gravid female mosquito was notably higher compared to that seen in control plates (Fig. 2). Bacteria isolates recovered from cage swab plates were assigned to the genus *Serratia*. Bacterial isolates from the body washes of gravid female mosquitoes were classified into genera *Serratia* and *Elizabethkingia*. *Bacillus* was the most common genus of bacteria found after interaction with a gravid female, and together with *Ornithinibacillus*, *Lysinibacillus*, and *Kroppenstedtia* constituted the genera exclusively associated with this experimental condition. Besides, the genera *Serratia* and *Paenibacillus* were also reported from female-exposed LB plates. The LB environmental controls showed bacterial growth in one of the two plates examined. This isolate was assigned to the genus *Paenibacillus*.

Culturable bacteria isolated from blood agar plates were represented by 36 isolates (Supplementary Table 2, Additional file 3). Bacteria belonged to three phyla, seven families, and six genera. Similarly, as observed with the LB medium, bacterial isolates recovered from blood agar plates exposed to a gravid female mosquito showed higher diversity compared with those from environmental controls (Fig. 2). Isolates obtained from the cage swab were members of the genus *Bacillus*. Blood agar plates on which the body wash of gravid females was plated generated four bacterial isolates assigned to genera *Elizabethkingia* and *Acinetobacter*. As with LB plates, *Bacillus* was the most common genus reported in blood agar plates visited by mosquitoes. The genera *Lysinibacillus* and *Staphylococcus* were exclusively associated with female visited samples. Besides, *Paenibacillus* and *Elizabethkingia* were also isolated in this condition. Finally, bacterial isolates recovered from the blood agar environmental controls were assigned to the genera *Bacillus* and *Paenibacillus*.

It is important to stress that *Serratia* (LB) and *Elizabethkingia* (blood agar) were the only genera shared between plates exposed to interaction with a gravid female and those from mosquito body washes.

### Changes in the bacterial profile of the breeding site

To verify whether females modify the bacterial profile of the water of a breeding site, an experiment comparing five treatments, differing in initial conditions, was designed (Fig. 1). Briefly, treatments 1 to 4 represented control conditions under which no female was allowed to oviposit, while T5 included a container in which a gravid female was allowed to oviposit on the water substrate for 24 h and then retired. T1 represented the same type of container, water, and sterilized food added 24 h later. T2 and T3 were initiated like T1 but had a controlled amount of mosquito eggs added manually together with the fish food. While for T2 the surface of eggs was sterilized to avoid the bacteria associated, eggs used for T3 were used *in natura*. Finally, for T4 a single female lacking access to water in the last 24 h was introduced to a similar experimental cage with a water container, but it was not gravid. In this case, we expected the female to visit the water (24 h) but show no oviposition. Water from each of the ten replicates per treatment was collected, their DNA was extracted and submitted for high throughput sequencing targeting the V4 region of the 16S rRNA gene that produced 20,425,105 reads from 50 water samples. After computational quality control, 16,896,903 reads were considered for taxonomic analysis. A data matrix was generated encompassing 532 ASVs. Nonetheless, the *HTSFilter* package identified a cutoff of 75 reads based on the Jaccard index. Therefore, all ASVs below this value were removed for downstream analysis. The total number of ASVs identified above the cut-off value was 159.

**Fig. 3:**
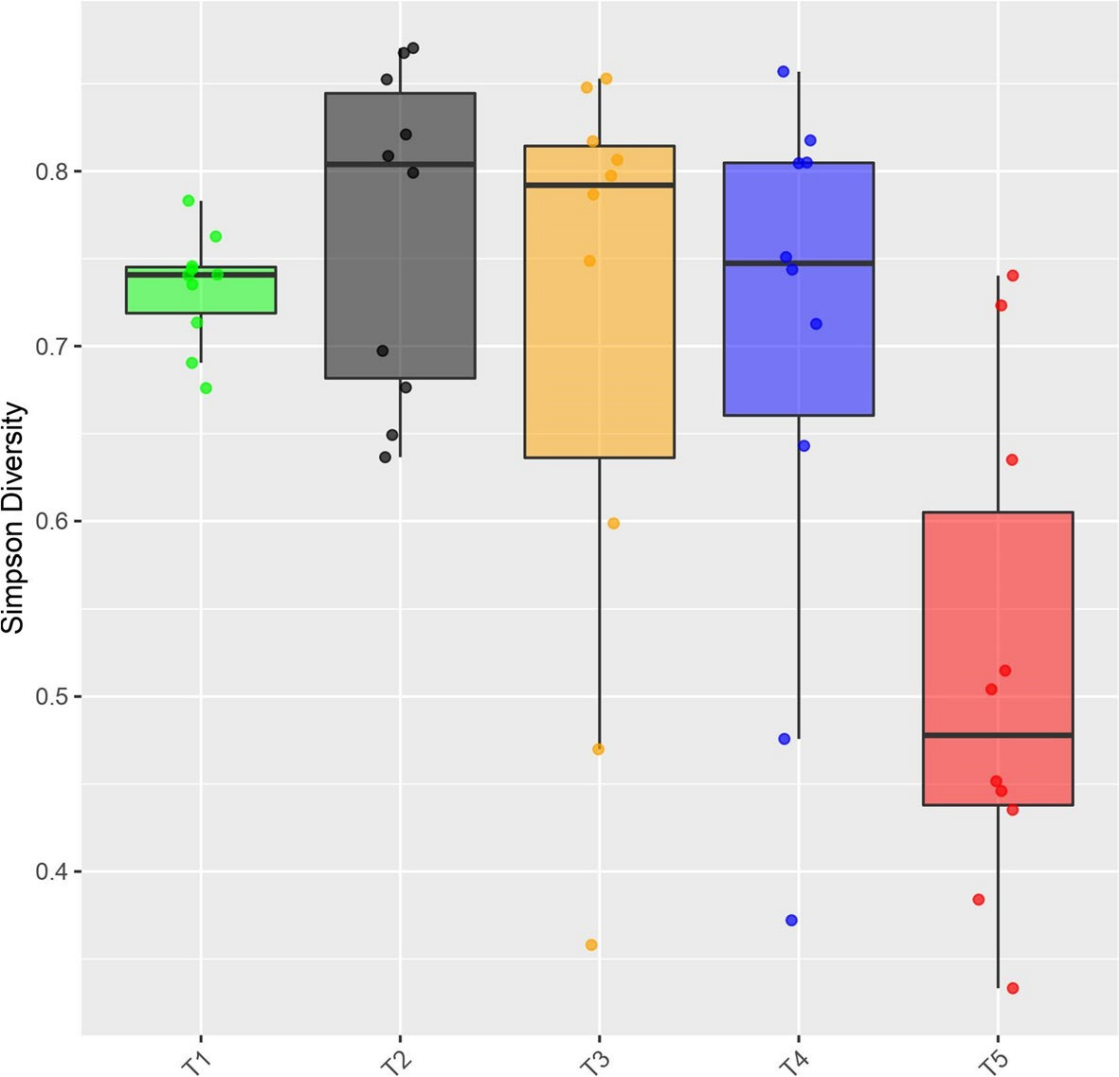
Alpha diversity of ASVs as a function of treatment. Comparison of Simpson’s Index of Diversity recorded for the different treatments using a boxplot (10 replicates and median). The alpha diversity was significantly different between experimental groups (Kruskal–Wallis, *P* = 0.01). Treatment 5 had a significantly lower Simpson index compared with the other four treatments. Treatment 1 (T1): environmental control (type I water plus sterilized food). Treatment 2 (T2): manually-deposited sterilized mosquito eggs. Treatment 3 (T3): manually-deposited non-sterilized eggs. Treatment 4 (T4): single sugar-fed females interacting with container water. Treatment 5 (T5): single gravid female (72 h post-blood-feeding) allowed to lay eggs

The alpha diversity was significantly different between experimental groups (Kruskal–Wallis, *P* = 0.01). Furthermore, the post hoc Dunn test identified that T5 had a significantly lower Simpson index compared with the other four treatments (Supplementary Table 1, Additional file 4). This is also depicted by the dominance of a particular bacterial taxon identified in the community composition (Additional file 5). Water samples belonging to T2 had the highest ASV diversity (mean Simpson index = 0.768), while T5 presented the lowest one (mean Simpson index = 0.527) (Fig. 3).

**Fig. 4:**
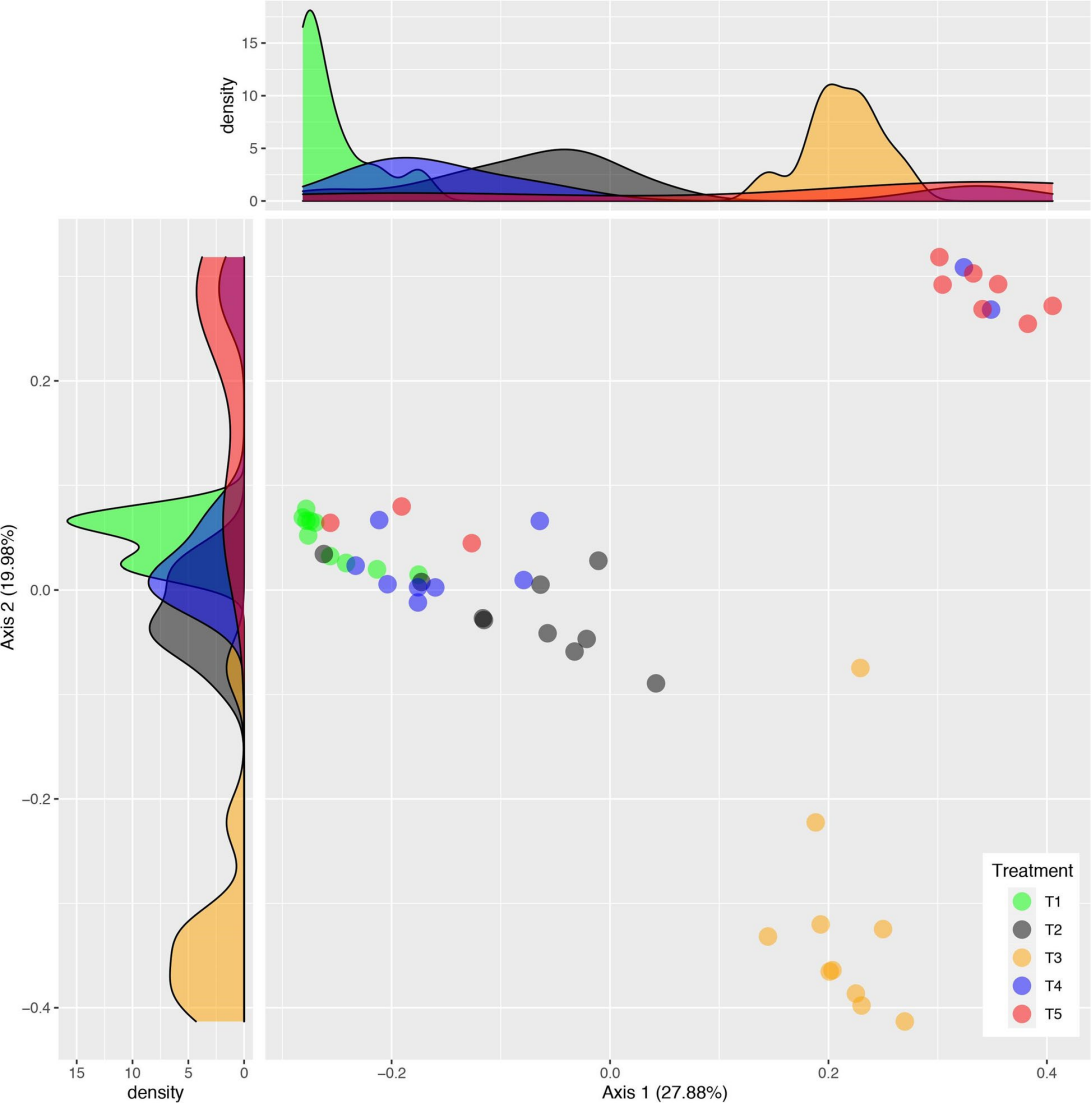
Beta diversity analysis. Principal coordinates analysis of Jensen-Shannon distances. Distinct clustering patterns for each experimental treatment and their corresponding replicates are represented by a color code. Axis 1 (27.88%) and Axis 2 (19.9%) show the percentage of variation explained. Treatment 1 (T1): environmental control (type I water plus sterilized food). Treatment 2 (T2): manually-deposited sterilized mosquito eggs. Treatment 3 (T3): manually-deposited non-sterilized eggs. Treatment 4 (T4): single sugar-fed females interacting with container water. Treatment 5 (T5): single gravid female (72 h post-blood-feeding) allowed to lay eggs

The Jensen-Shannon divergence metric was used to compare ASV diversity among treatments (Additional file 6). The PCoA captured around 48% of the variation in Jensen-Shannon distance along the two chosen axes (PCo_1_ and PCo_2_) represented in Fig. 4. A comparison of the bacterial communities associated with each treatment showed distinct clustering patterns. Samples belonging to T3 and T5 displayed higher inter-treatment variability clustering bottom and top right, respectively (Fig. 4).

The PERMANOVA revealed that the presence of eggs (*R*^2^ = 0.206, df = 2, *P* = 0.001), female interaction with water (*R*^2^ = 0.103, df = 1, *P* = 0.001), and the interaction of these two variables (*R*^2^ = 0.092, df = 1, *P* = 0.001) explain 40% of the variance in bacterial composition over the groups of samples. Additionally, the pairwise PERMANOVA confirmed that all groups differ significantly from each other in terms of beta diversity, suggesting that there are consistent bacterial signature profiles for each condition (Supplementary Table 2, Additional file 4).

The RF model selected 10 ASVs as the most important features for each variable explored — female interaction with water and egg presence (Tables 1 and 2). The bacterial signatures modeled by RF had robust prediction performances supported by their high AUC (Area Under de Curve) values (Supplementary Fig. 1, Additional file 4).

**Table 1:** ASVs predictive of female interaction with water. The 10 most important discriminating ASVs identified by RF when a female *Aedes aegypti* interacted with water

ASV ID	Family	Species	MeanDecreaseGini
sq2	*Weeksellaceae*	*Elizabethkingia anophelis*	2.7596470
sq81	*Xanthomonadaceae*	*Stenotrophomonas maltophilia*	1.8039565
sq12	*Rhizobiaceae*	*Agrobacterium radiobacter*	1.0807458
sq10	*Sphingomonadaceae*	unclassified_*Novosphingobium*	1.0666743
sq23	*Burkholderiaceae*	unclassified_*Pseudacidovorax*	0.9449007
sq21	*Enterobacteriaceae*	unclassified_*Enterobacter*	0.8477820
sq6	*Moraxellaceae*	unclassified_*Acinetobacter*	0.8216041
sq28	*Xanthomonadaceae*	*Stenotrophomonas maltophilia*	0.7735763
sq49	*Burkholderiaceae*	*Herbaspirillum huttiense*	0.6960721
sq9	*Pseudomonadaceae*	*Pseudomonas putida*	0.7169478

**Table 2:** ASVs predictive of egg presence. The 10 most important discriminating ASVs identified by RF when *Aedes aegypti* eggs were present in water

ASV ID	Family	Species	MeanDecreaseGini
sq31	*Burkholderiaceae*	unclassified_*Pelomonas*	2.3970572
sq45	*Xanthobacteraceae*	*Bradyrhizobium japonicum*	2.3434994
sq1	*Caulobacteraceae*	*Caulobacter vibrioides*	2.2397369
sq11	*Burkholderiaceae*	*Ralstonia insidiosa*	1.6738255
sq67	*Nocardiaceae*	*Rhodococcus erythropolis*	1.3978588
sq18	*Sphingomonadaceae*	unclassified_*Sphingomonas*	1.3206670
sq42	*Xanthobacteraceae*	*Afipia genosp.*	1.2906533
sq23	*Burkholderiaceae*	unclassified_*Pseudacidovorax*	0.9296086
sq2	*Weeksellaceae*	*Elizabethkingia anophelis*	0.9353752
sq10	*Sphingomonadaceae*	unclassified_*Novosphingobium*	0.8482134

On the other hand, indicator species analysis identified ASVs considered to be specific microbial features associated with the act of oviposition and larval development, i.e., T5. Seven ASVs were pinpointed as oviposition-indicating species as they possess significant fidelity and predictive value towards the ecological conditions represented in this niche/treatment (Table 3). Indicator species were assigned to the following taxa*: Leifsonia soli*, *Elizabethkingia anophelis*, *Paenibacillus polymyxa*, *Stenotrophomonas maltophilia*, *Elizabethkingia*, *Methylobacterium*, and *Elizabethkingia meningoseptica*.

**Table 3:** Indicator species analysis. ASVs considered features of oviposition activity (treatment 5)

ASV ID	Bacterial taxa	Specificity	Fidelity	*Indicstat*	*P*-value
sq44	*Leifsonia soli*	0.8540	0.9000	0.877	0.001
sq2	*Elizabethkingia anophelis*	0.7274	1.0000	0.853	0.001
sq75	*Paenibacillus polymyxa*	0.7911	0.8000	0.796	0.001
sq81	*Stenotrophomonas maltophilia*	0.5068	0.9000	0.675	0.004
sq86	*Elizabethkingia*	0.7355	0.6000	0.664	0.001
sq85	*Methylobacterium*	0.7138	0.6000	0.654	0.034
sq143	*Elizabethkingia meningoseptica*	0.9362	0.4000	0.612	0.003

### *Aedes aegypti* exhibits faster development in the presence of *Elizabethkingia*

**Fig. 5:**
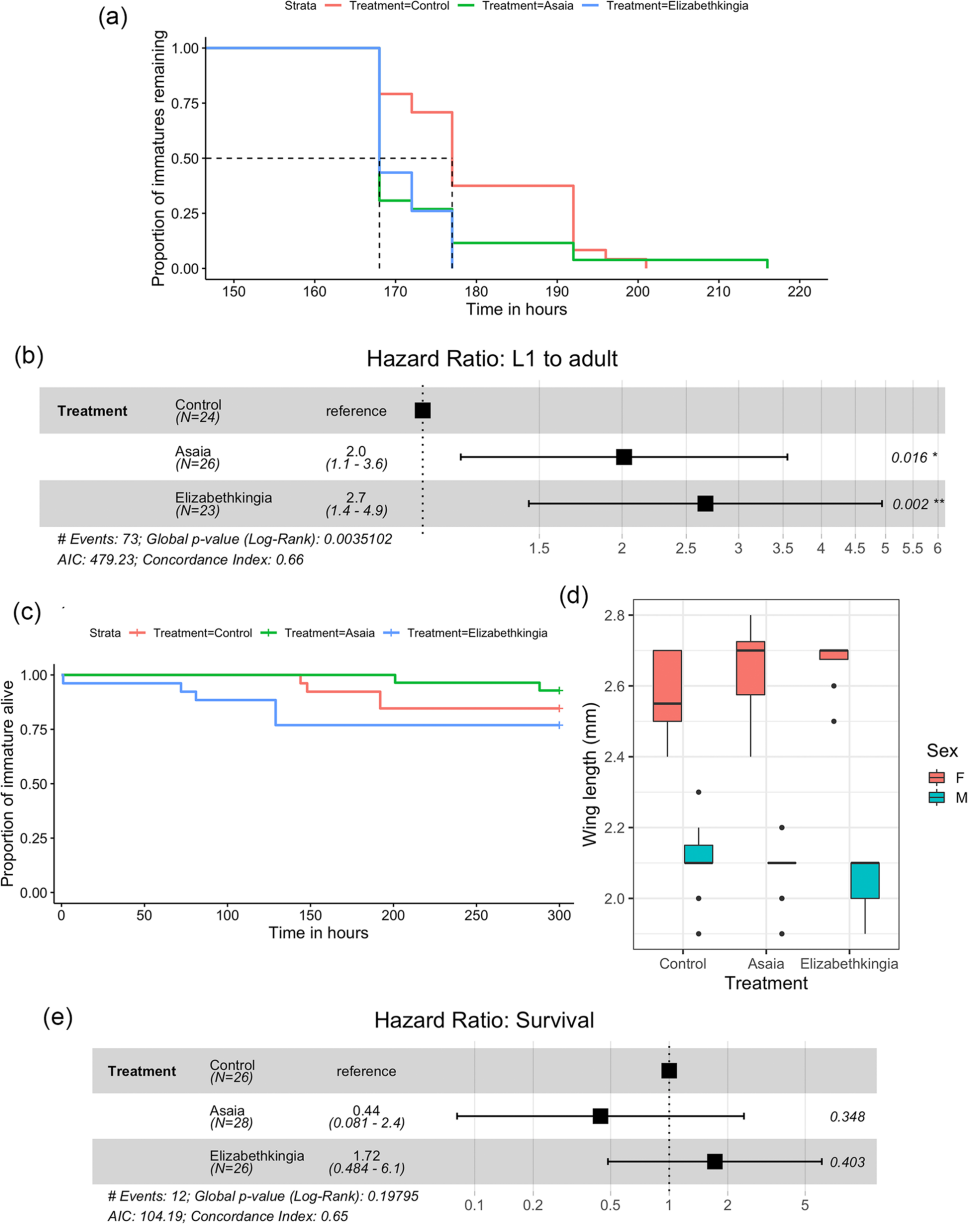
Duration of larval development (L1 to adult) of *Aedes aegypti* exposed to bacteria of the genera *Asaia* or *Elizabethkingia* and the control group. The dashed lines indicate median developmental time (**a**). Hazard ratios and 95% confidence intervals associated with *Asaia* or *Elizabethkingia* exposure were estimated using Cox Proportional-Hazard models with larval development as a dependent variable. Black squares represent the hazard ratios and the horizontal bars extend from the lower limit to the upper limit of the 95% confidence intervals of the hazard ratios (**b**)

Median total immature development (L1 to adult) took 177 h for the control, and 168 h for the *Asaia* and *Elizabethkingia*-exposed larvae (Fig. 5a and Supplementary Table 3, Additional file 4). The exposure to both bacteria significantly reduced the total immature development time when compared to the control group (Fig. 5b). For *Asaia*-exposed larvae, the HR for total development was 2.0 (95% confidence interval: 1.1–3.6, Fig. 5) with detectable differences only in the pupal stage (Additional file 7e). The effect of *Elizabethkingia* was more prominent with an HR of 2.7 (95% confidence interval: 1.4–4.9, Fig. 5) and detectable differences only in the L1 stage (Additional file 7a). Regarding survival, control, *Asaia* and *Elizabethkingia* exposed specimens presented 15, 8, and 23% of mortality, respectively. The effect of bacteria exposure on immature survival was not statistically significant (global *p*-value from log-rank = 0.20, non-significant HRs, Fig. 5d and e) nor wing length (KW chi-squared for males = 3.95, *P* = 0.14; KW chi-squared for females = 2.71, *P* = 0.25, Fig. 5c).

The diversity of culturable microbiota was variable between the three groups, with only the *Bacillus* genus being ubiquitous to all conditions (Additional file 8). *Asaia* was not isolated from any of them, while *Elizabethkingia* was recovered from the midgut of larvae exposed to it.

## Discussion

This study has explored the hypothesis that ovipositing females shape the microbial consortium of the aquatic niche of the breeding site to promote larval fitness. Our results showed that gravid females mechanically transmit viable and culturable bacteria already reported as mosquito symbionts. We then demonstrated that the act of oviposition promoted a significant decrease in the bacterial diversity found in breeding sites. Furthermore, this was associated with a specific bacterial profile which included a series of indicator taxa linked to female oviposition. We finally presented evidence that demonstrates that one of these taxa, i.e., *Elizabethkingia*, was able to accelerate larval development. Altogether, these results seem indicative of female-induced niche construction in *Aedes aegypti* breeding sites.

Experiments showing mechanical transmission from females to plates confirm that gravid mosquitoes can inoculate bacteria. *Bacillus* was predominant on plates visited by gravid females (for both blood agar and LB media). This bacterial taxon has been already identified in stable association with larvae [47] and adult *Ae. aegypti* [48]. Interestingly, other bacteria inoculated were frequently reported as key members of mosquito microbiota, e.g., *Elizabethkingia* and *Serratia* [40], both being vertically, horizontally, and transstadially transmitted [20, 22, 49]. It should be noted that moist agar plates eventually induced oviposition. Therefore, we suggest that while exploring a tentative oviposition site, gravid females inoculate the substrate with bacterial partners that according to our experiments support offspring development.

Next, we evaluated whether gravid females influence the bacterial communities of water-holding containers and showed that the act of oviposition significantly decreased their diversity. As the Simpson index is a dominance metric [50], we suggest that an uneven microcosm (dominated by the most abundant taxa) represents an advantageous scenario for larvae because not all microbes are beneficial. The latter could either be due to their pathogenicity or because they do not fulfill key functions in the host-microbe network [51]. An ecosystem with a low-diversity microbial community but high-fidelity microbial partners would favor the establishment of specific mutualistic interactions [52]. The above is congruent with recent observations made by Martinson and Strand [53], who highlighted the successful development of *Ae. aegypti* larvae in low diversity (gnotobiotic communities) breeding sites given certain dietary conditions were met. The decrease in community evenness seen in T5 can be considered a hallmark sign of susceptibility towards the establishment of an invading organism in the community, i.e. the larvae [54, 55].

Regarding beta diversity, the ordination analysis showed how the community structures diverge among treatments, particularly highlighting differences driven by factors other than oviposition. As each treatment represented potential sources of microbial inocula, it is relevant to highlight how the single unit of the natural egg-laying plus larval development (T5) predominantly presented a profile that diverged from the others in the ordination space. Curiously, non-sterile eggs (T3), representing the outcome of the general procedure for rearing mosquitoes in insectary conditions, produced a clearly different profile. It is also relevant to observe that surface-sterilized eggs manually deposited in the water (T2) presented a profile resembling that of the control and non-gravid female-water interactions. We propose that stereotypical female behaviors expressed while ovipositing (e.g., grooming, tasting, defecating) would be fundamental to generate the unique bacterial profile seen for treatment 5. This is reinforced by the fact that larval presence did not lead to similar profiles of beta diversity in other treatments.

Three out of seven ASVs pointed out by the indicator species analysis belonged to the *Elizabethkingia* genus which was shown to be capable of inhibiting *Pseudomonas*, another mosquito-associated bacterium, via an antimicrobial independent mechanism [56]. In addition, *Elizabethkingia* has broad antibiotic resistance because of a large number of genes encoding efflux pumps and β-lactamases present in its genome [57]. Bacteria use diverse mechanisms to compete with other members of the microbial community [51], and based on the above information, *Elizabethkingia* likely disturbed the bacterial consortium by eliminating competitors at the breeding site, as our experiment seems to suggest.

Finally, we evaluated whether *Elizabethkingia* and *Asaia* influenced larval developmental time, survival, and adult size in *Ae. aegypti*. This was intended to compare the impact of this oviposition-indicating taxon with that of *Asaia*, another bacterium reported in mosquito microbiota but not found in our breeding sites. *Elizabethkingia* significantly speeded up development and colonized the larval midgut suggesting a facilitated interaction between them. Indeed, reducing mosquito larval development time might increase the probability of reaching adulthood [58]. In this context, the presence of *Elizabethkingia* in breeding water and larval midguts has likely aided metabolic activities, providing nutrients or metabolites that stimulate faster larval development and/or represented an additional source of food [16, 49]. Taken together our results suggest that females can spike breeding sites with this symbiotic bacterium to support offspring fitness, which could be interpreted as a form of niche construction. However, it is important to notice that this effect on larval development was not exclusive to *Elizabethkingia* since *Asaia* also accelerated the larval development of *Ae. aegypti*, as previously observed for *Anopheles gambiae* [41, 42].

We revealed an ecological and functional connection between egg-laying activity, the bacterial communities in *Ae. aegypti* breeding sites, a key symbiotic bacterial taxon, and the speed of larval development by using a set of different experimental and analytical methodologies, including testing field-originated bacterial symbionts. Other microorganisms have individual or combined positive impacts on the larval development of *Ae. aegypti* [53, 59]. As such, we concur with the concept that these effects most likely will be understood from a community ecology perspective [40]. Other indicator taxa from our set, as well as other microorganisms and their interactions, could be the driving forces detected in the compositional profile of T5 and be key to the success of the breeding sites.

## Conclusions

Niche construction theory recognizes that organisms can modify both biotic and abiotic components of their environments. This process is an outcome of their activities, metabolism, and choices, and its main consequence is to increase survival probabilities [26, 60]. Altogether, our findings suggest that niche construction may represent a strategy used by female *Ae. aegypti* to disseminate symbiotic bacteria through egg-laying to grant proper environments for their progeny. As stressed by Schwab and collaborators [29], our results are in agreement with niche construction theory criteria: a substantial environment modification was detected (bacterial community diversity), and positive fitness/developmental consequences were measured when a biomarker taxon was used as an effector. These findings provide solid grounds to build upon and improve our knowledge of how endo and ecto microbiomes may be critical when addressing their links to host phenotypes through the lens of niche construction theory. Other layers of information may be relevant to improve this take, as the metabolite profile of breeding sites also reflects the act of oviposition and development [61]. Besides, oviposition by several females responding to bacterially-emitted volatile organic compounds may contribute other symbionts to this community-shaping process ongoing in breeding sites [62]. Disentangling whether and how individual microorganisms, or their networks, exert effects on mosquito life-history traits is a growing field of study benefiting from the synergy of microbiology, ecology, physiology, and computational biology.

## Supplementary Information

**Additional file 1: Supplementary Table 1.** Number of eggs laid by each *Aedes aegypti* female in treatment 5.

**Additional file 2: Supplementary Figure 1.** Negative controls performed during sample processing did not generate any amplicons during the quality control carried out by the sequencing facility.

**Additional file 3: Supplementary Table 1.** Bacterial taxonomic affiliation of isolates recovered from LB agar plates. Supplementary Table 2. Bacterial taxonomic affiliation of isolates recovered from blood agar plates.

**Additional file 4: Supplementary Table 1.** Dunn test comparing alpha diversity between treatments. P values adjusted with the Benjamini-Hochberg method are shown. **Supplementary Table 2.** Pairwise PERMANOVA showing between-group shifts in bacterial signature profile when comparing the beta diversity among treatments. P values for each comparison are shown. Supplementary Figure 1. ROC curves for female interaction and eggs presence prediction. The point indicates the best cutoff value from the prediction probability to optimize Sensitivity and Specificity. The confidence levels reflect a 95% confidence interval. **Supplementary Table 3.** Median, mean and standard deviation of instar duration of immature stages of *Aedes aegypti*, wing size, and survival exposed to *Asaia* and *Elizabethkingia* bacteria.

**Additional file 5: Supplementary Figure 1.** Bacterial community composition at order level for each water sample belonging to five different treatments.

**Additional file 6: Supplementary Figure 1.** Heatmap of the Jensen-Shannon distances between the five treatments.

**Additional file 7: Supplementary Figure 1.** Duration of all larval instars and pupal phase of *Aedes aegypti* exposed to bacteria of the genera *Asaia* or *Elizabethkingia* and the control group. The dashed lines in the graphs indicate median developmental time. Hazard ratios and 95% confidence intervals associated with *Asaia* or *Elizabethkingia* exposure were estimated using Cox Proportional-Hazard models with developmental time as the dependent variable. Black squares represent the hazard ratios and the horizontal bars extend from the lower limit to the upper limit of the 95% confidence intervals of the hazard ratios.

**Additional file 8: Supplementary Table 1.** Bacteria genera isolated from *Aedes aegypti* exposed to bacteria of the genera *Asaia* or *Elizabethkingia* and the control group.

## Data Availability

Raw sequence data are available at the European Nucleotide Archive, project number: PRJEB51063 [63].

## Abbreviations

LB: Lysogeny Broth
PBS: Phosphate-buffered saline (PBS)
16S rRNA: 16S ribosomal RNA
PCR: Polymerase chain reaction
T1: Treatment 1
T2: Treatment 2
T3: Treatment 3
T4: Treatment 4
T5: Treatment 5
ASV: Amplicon sequence variant
PCoA: Principal coordinates analysis
PERMANOVA: Permutational Multivariate Analysis of Variance
RF: Random Forest
L1: Larval stage 1
L2: Larval stage 2
L3: Larval stage 3
L4: Larval stage 4
HR: Hazard ratio
AUC: Area under the curve
ROC: Receiver operating characteristic curve
AIC: Akaike information criterion
SD: Standard deviation
