# Supplementary material for: Egg-laying by female Aedes aegypti shapes the bacterial communities of breeding sites
Source: BMC Biol. 2023 Apr 26;21:97. doi: 10.1186/s12915-023-01605-2 (PMC10134544; doi:10.1186/s12915-023-01605-2)
Supplement: Supplementary file 1 — Additional file 1: Supplementary Table 1. Number of eggs laid by each Aedes aegypti female in treatment 5. [file 12915_2023_1605_MOESM1_ESM.pdf]

Additional file 1

Supplementary table 1. Number of eggs laid by each individual *Aedes aegypti* female in treatment 5.

**Gravid females**	**Number of eggs**
Female 1	3
Female 2	59
Female 3	78
Female 4	33
Female 5	90
Female 6	30
Female 7	53
Female 8	84
Female 7	53
Female 8	84
Female 9	4
Female 10	78
**Mean number of eggs**	51.2
