# Supplementary material for: Egg-laying by female Aedes aegypti shapes the bacterial communities of breeding sites
Source: BMC Biol. 2023 Apr 26;21:97. doi: 10.1186/s12915-023-01605-2 (PMC10134544; doi:10.1186/s12915-023-01605-2)
Supplement: Supplementary file 2 — Additional file 2: Supplementary Figure 1. Negative controls performed during sample processing did not generate any amplicons during the quality control carried out by the sequencing facility. [file 12915_2023_1605_MOESM2_ESM.pdf]

Additional file 2

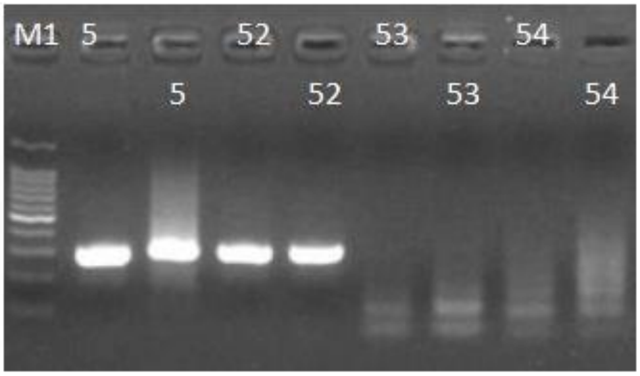

Note:
M1,Trans 100bp ladder
samples ranged in the order of upper table (All loaded 3μL)

Supplementary figure 1. Negative controls performed during sample processing did not generate any amplicons (wells 53 and 54) during the quality control carried out by the sequencing facility (gel generated by Novogene).
