# Supplementary material for: Egg-laying by female Aedes aegypti shapes the bacterial communities of breeding sites
Source: BMC Biol. 2023 Apr 26;21:97. doi: 10.1186/s12915-023-01605-2 (PMC10134544; doi:10.1186/s12915-023-01605-2)
Supplement: Supplementary file 3 — Additional file 3: Supplementary Table 1. Bacterial taxonomic affiliation of isolates recovered from LB agar plates. Supplementary Table 2. Bacterial taxonomic affiliation of isolates recovered from blood agar plates. [file 12915_2023_1605_MOESM3_ESM.pdf]

Additional file 3

Supplementary table 1. Bacterial taxonomic affiliation of isolates recovered from LB agar plates.

Plate where the isolate came from	Isolate	No. of bases used to establish identity	Phylum	Presumable genus (% sequence identity)
Cage swab	L1	916	Proteobacteria	*Serratia* (100)
Cage swab	L2	---	---	unidentified
Cage swab	L3	885	Proteobacteria	*Serratia* (99.7)
Cage swab	L4	914	Proteobacteria	*Serratia* (99.6)
Body wash 1	L5	916	Proteobacteria	*Serratia* (99.7)
Body wash 2	L6	709	Bacteroidetes	*Elizabethkingia* (95.4)
Body wash 2	L7	783	Proteobacteria	*Serratia* (94.9)
Female 1	L8	1339	Firmicutes	*Bacillus* (95.8)
Female 1	L9	828	Firmicutes	*Bacillus* (99.5)
Female 1	L10	840	Firmicutes	*Bacillus* (99.5)
Female 1	L11	836	Firmicutes	*Ornithinibacillus* (98.3)
Female 2	L12	1328	Firmicutes	*Bacillus* (98.8)
Female 2	L13	1421	Firmicutes	*Bacillus* (99.9)
Female 3	L14	846	Proteobacteria	*Serratia* (99.7)
Female 3	L15	910	Firmicutes	*Paenibacillus* (98.7)
Female 4	L16	783	Firmicutes	*Bacillus* (99.6)
Female 4	L17	1372	Firmicutes	*Bacillus* (98.5)
Female 4	L18	1333	Firmicutes	*Lysinibacillus* (97.6)
Female 4	L19	1330	Firmicutes	*Bacillus* (98.5)
Female 4	L20	1340	Firmicutes	*Bacillus* (98.2)
Female 4	L21	829	Firmicutes	*Bacillus* (97.9)
Female 4	L22	1376	Firmicutes	*Kroppenstedtia* (92.4)
Female 5	L23	832	Firmicutes	*Bacillus* (99.2)
Female 5	L24	833	Firmicutes	*Bacillus* (99.6)
Female 5	L25	823	Firmicutes	*Bacillus* (99.7)
Female 5	L26	1367	Firmicutes	*Bacillus* (99.9)
Female 5	L27	1373	Firmicutes	*Bacillus* (98.4)
Control 1	L28	1373	Firmicutes	*Paenibacillus* (99.8)

Supplementary table 2. Bacterial taxonomic affiliation of isolates recovered from blood agar plates.

**Plate where the isolate came from**	**Isolate**	**No. of bases used to establish identity**	**Phylum**	**Presumable genus (% sequence identity)**
Cage swab	B1	1361	Firmicutes	*Bacillus* (100)
Cage swab	B2	1332	Firmicutes	*Bacillus* (100)
Cage swab	B3	1329	Firmicutes	*Bacillus* (100)
Cage swab	B4	1298	Firmicutes	*Bacillus* (99.7)
Body wash 1	B5	1412	Bacteroidetes	*Elizabethkingia* (99.7)
Body wash 1	B6	1338	Proteobacteria	*Acinetobacter* (98)
Body wash 2	B7	794	Bacteroidetes	*Elizabethkingia* (99.8)
Body wash 2	B8	---	---	unidentified
Female 1	B9	1355	Firmicutes	*Paenibacillus* (100)
Female 1	B10	1390	Firmicutes	*Bacillus* (99.9)
Female 1	B11	1376	Firmicutes	*Bacillus* (99.9)
Female 1	B12	1348	Firmicutes	*Bacillus* (99.6)
Female 2	B13	1423	Firmicutes	*Bacillus* (99.9)
Female 2	B14	1418	Firmicutes	*Bacillus* (99.9)
Female 2	B15	1031	Firmicutes	*Bacillus* (99.9)
Female 2	B16	1364	Firmicutes	*Bacillus* (97.3)
Female 3	B17	1367	Firmicutes	*Bacillus* (99.6)
Female 3	B18	1374	Firmicutes	*Lysinibacillus* (97.8)
Female 3	B19	1369	Firmicutes	*Bacillus* (99.8)
Female 3	B20	937	Firmicutes	*Bacillus* (99.7)
Female 3	B21	1366	Firmicutes	*Bacillus* (99.9)
Female 3	B22	912	Firmicutes	*Bacillus* (99.7)
Female 4	B23	1417	Firmicutes	*Bacillus* (99.8)
Female 4	B24	1456	Firmicutes	*Paenibacillus* (99.7)
Female 4	B25	1445	Firmicutes	*Bacillus* (100)
Female 4	B26	922	Firmicutes	*Staphylococcus* (99.8)
Female 4	B27	1359	Firmicutes	*Bacillus* (99.6)
Female 5	B28	954	Firmicutes	*Bacillus* (99.4)
Female 5	B29	1356	Firmicutes	*Bacillus* (99.7)
Female 5	B30	814	Firmicutes	*Paenibacillus* (97.8)
Female 5	B31	1358	Firmicutes	*Bacillus* (99.6)
Female 5	B32	1411	Firmicutes	*Bacillus* (99.8)
Female 5	B33	879	Bacteroidetes	*Elizabethkingia* (97.8)
Female 5	B34	1334	Firmicutes	*Bacillus* (99.8)
Control 1	B35	1310	Firmicutes	*Paenibacillus* (99.3)
Control 2	B36	804	Firmicutes	*Bacillus* (99.6)
