# Supplementary material for: Egg-laying by female Aedes aegypti shapes the bacterial communities of breeding sites
Source: BMC Biol. 2023 Apr 26;21:97. doi: 10.1186/s12915-023-01605-2 (PMC10134544; doi:10.1186/s12915-023-01605-2)
Supplement: Supplementary file 4 — Additional file 4: Supplementary Table 1. Dunn test comparing alpha diversity between treatments. P values adjusted with the Benjamini-Hochberg method are shown. Supplementary Table 2. Pairwise PERMANOVA showing between-group shifts in bacterial signature profile when comparing the beta diversity among treatments. P values for each comparison are shown. Supplementary Figure 1. ROC curves for female interaction and eggs presence prediction. The point indicates the best cutoff value from the prediction probability to optimize Sensitivity and Specificity. The confidence levels reflect a 95% confidence interval. Supplementary Table 3. Median, mean and standard deviation of instar duration of immature stages of Aedes aegypti, wing size, and survival exposed to Asaia and Elizabethkingia bacteria. [file 12915_2023_1605_MOESM4_ESM.pdf]

Additional file 4

Supplementary table 1. Dunn test comparing alpha diversity between treatments. P values adjusted with the Benjamini-Hochberg method are shown.

	Treatment 2	Treatment 3	Treatment 4	Treatment 5
Treatment 1	0.2563	0.3565	0.4090	0.0209
Treatment 2	–	0.4074	0.3045	0.0021
Treatment 3	–	–	0.4088	0.0077
Treatment 4	–	–	–	0.0145

Supplementary table 2. Pairwise PERMANOVA showing between-group shifts in bacterial signature profile when comparing the beta diversity among treatments. P values for each comparison are shown.

	Treatment 2	Treatment 3	Treatment 4	Treatment 5
Treatment 1	0.001	0.001	0.001	0.001
Treatment 2	–	0.001	0.001	0.001
Treatment 3	–	–	0.001	0.001
Treatment 4	–	–	–	0.004

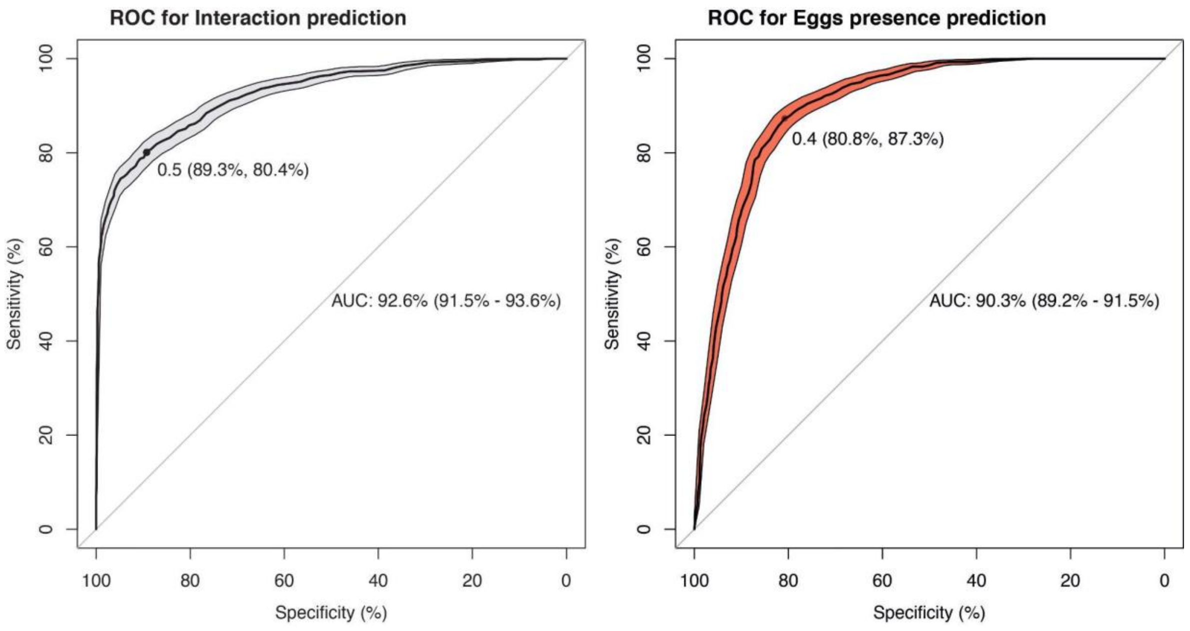

Supplementary figure 1. ROC curves for female interaction (left) and eggs presence (right) prediction. The point indicates the best cutoff value from the prediction probability to optimize Sensitivity and Specificity. The confidence levels reflect a 95% confidence interval.

Supplementary table 3. Median, mean and standard deviation (SD) of instar duration (hours) of immature stages of *Aedes aegypti* (Paea strain), wing size, and survival (95% confidence interval) exposed to *Asaia* and *Elizabethkingia* bacteria.

	**Control**			**+*Asaia***			**+*Elizabethkingia***		
	**Median**	**Mean**	**SD**	**Median**	**Mean**	**SD**	**Median**	**Mean**	**SD**
**L1 (h)**	33	40.28	7.60	33	37.11	9.13	33	32.71	5.27
**L2 (h)**	19	15.77	7.05	19	18.63	7.57	19	18.03	3.14
**L3 (h)**	24	24.32	3.55	24	24.30	4.89	24	23.73	1.46
**L4 (h)**	48	50.11	7.88	48	54.11	28.03	48	47.30	5.26
**Pupa (h)**	50	52.37	5.61	48	46.84	6.51	48	49.43	7.08
**L1 to adult (h)**	177	180.87	10.64	168	173.23	11.10	168	171.04	3.91
**Wing length**	2.2	2.27	0.25	2.1	2.27	0.28	2.1	2.27	0.31
**Sex ratio (M:F)**	1.9:1			2.1:1			1.7:1		
**Survival (95% CI)**	0.85 (0.72 - 1.0)			0.92 (0.84 - 1)			0.77 (0.62 - 0.95)		
