# Supplementary material for: Egg-laying by female Aedes aegypti shapes the bacterial communities of breeding sites
Source: BMC Biol. 2023 Apr 26;21:97. doi: 10.1186/s12915-023-01605-2 (PMC10134544; doi:10.1186/s12915-023-01605-2)
Supplement: Supplementary file 5 — Additional file 5: Supplementary Figure 1. Bacterial community composition at order level for each water sample belonging to five different treatments. [file 12915_2023_1605_MOESM5_ESM.pdf]

Additional file 5

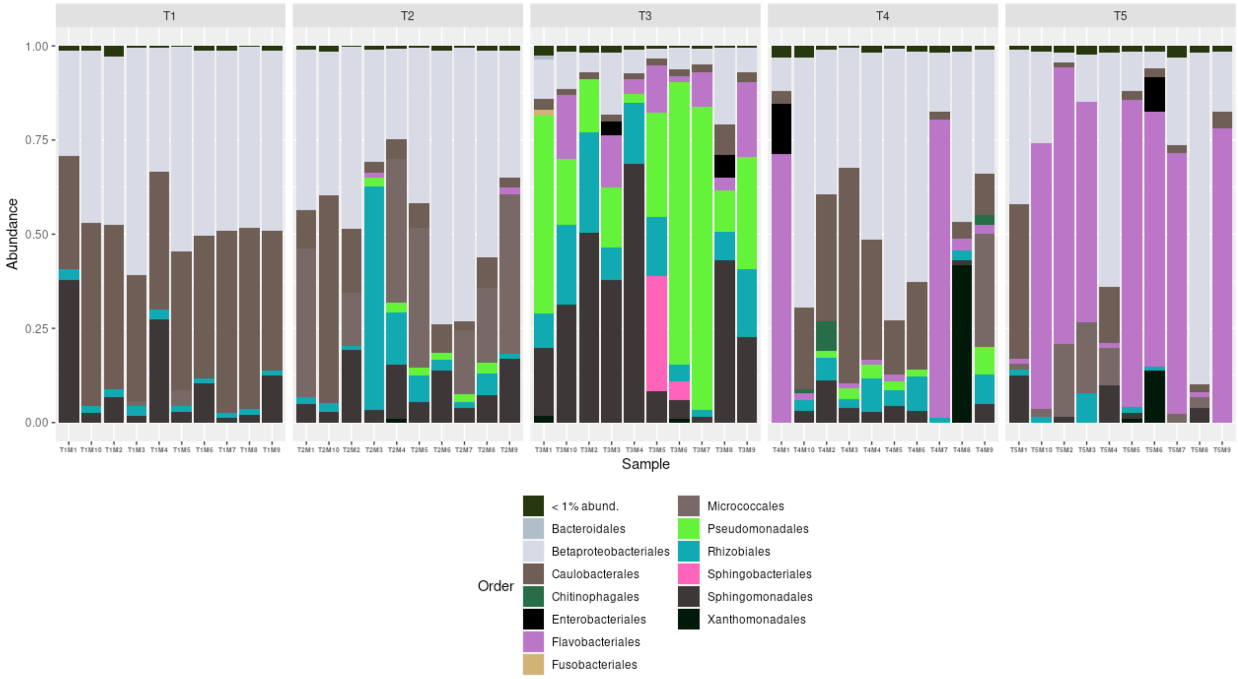

Supplementary figure 1. Bacterial community composition at order level for each water sample belonging to five different treatments.
