# Supplementary material for: Egg-laying by female Aedes aegypti shapes the bacterial communities of breeding sites
Source: BMC Biol. 2023 Apr 26;21:97. doi: 10.1186/s12915-023-01605-2 (PMC10134544; doi:10.1186/s12915-023-01605-2)
Supplement: Supplementary file 6 — Additional file 6: Supplementary Figure 1. Heatmap of the Jensen-Shannon distances between the five treatments. [file 12915_2023_1605_MOESM6_ESM.pdf]

Additional file 6

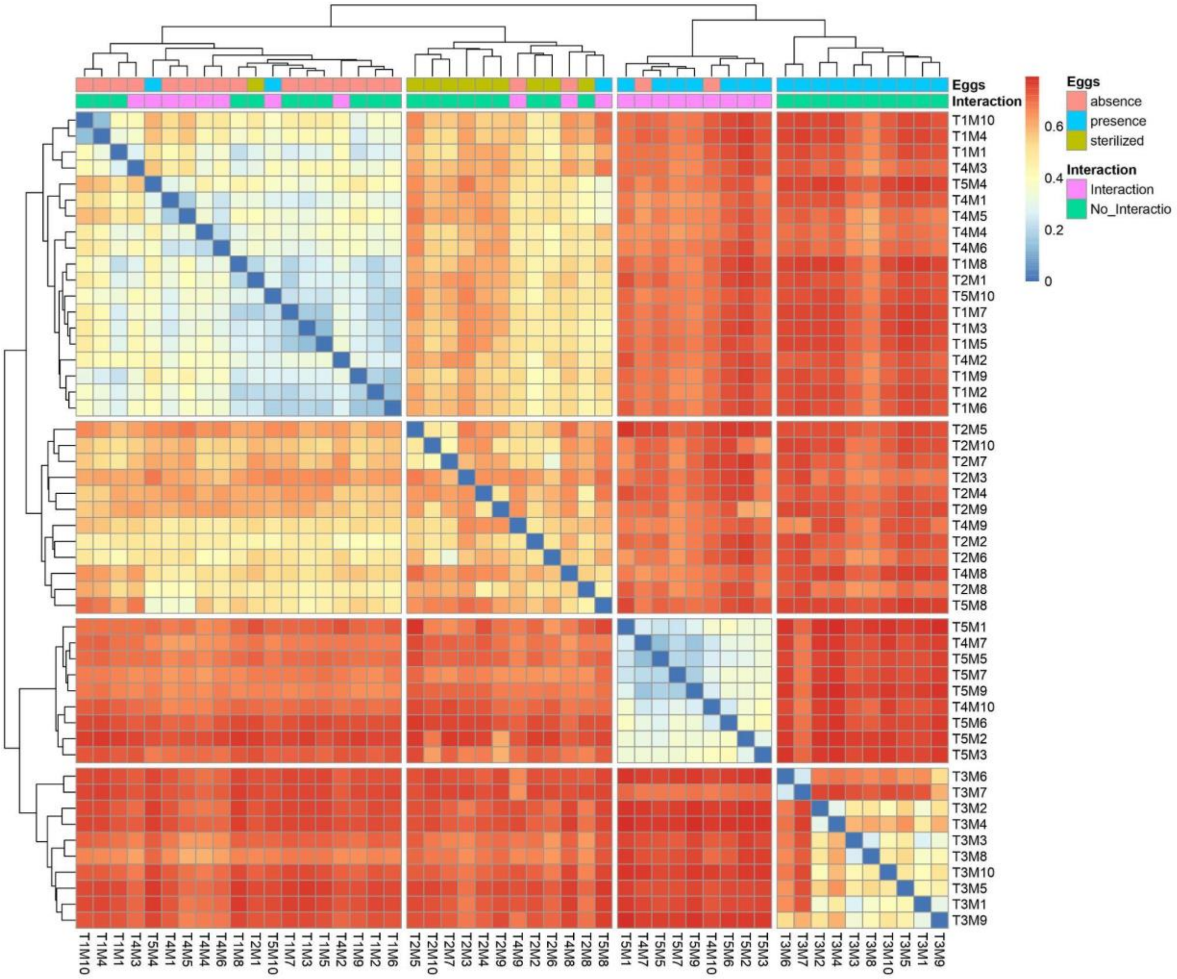

Supplementary figure 1. Heatmap of the Jensen-Shannon distances between the five treatments.
