# Supplementary material for: Egg-laying by female Aedes aegypti shapes the bacterial communities of breeding sites
Source: BMC Biol. 2023 Apr 26;21:97. doi: 10.1186/s12915-023-01605-2 (PMC10134544; doi:10.1186/s12915-023-01605-2)
Supplement: Supplementary file 7 — Additional file 7: Supplementary Figure 1. Duration of all larval instars and pupal phase of Aedes aegypti exposed to bacteria of the genera Asaia or Elizabethkingia and the control group. The dashed lines in the graphs indicate median developmental time. Hazard ratios and 95% confidence intervals associated with Asaia or Elizabethkingia exposure were estimated using Cox Proportional-Hazard models with developmental time as the dependent variable. Black squares represent the hazard ratios and the horizontal bars extend from the lower limit to the upper limit of the 95% confidence intervals of the hazard ratios. [file 12915_2023_1605_MOESM7_ESM.pdf]

Additional file 7

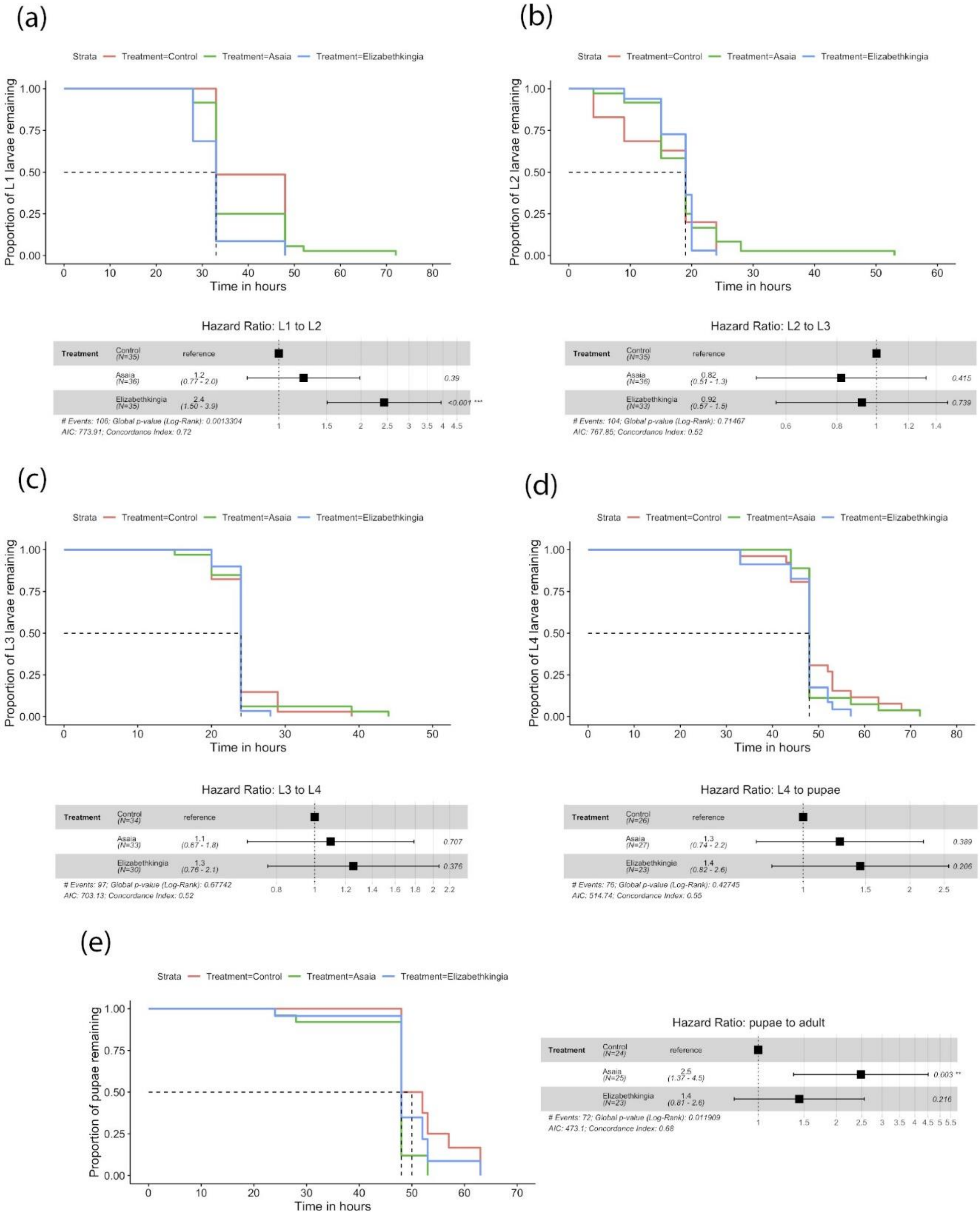

Supplementary figure 1. Duration of all larval instars (a to d) and pupal phase (e) of *Aedes aegypti* exposed to bacteria of the genera *Asaia* or *Elizabethkingia* and the control group.

The dashed lines in the graphs indicate median developmental time. Hazard ratios and 95% confidence intervals associated with *Asaia* or *Elizabethkingia* exposure were estimated using Cox Proportional-Hazard models with developmental time as the dependent variable. Black squares represent the hazard ratios and the horizontal bars extend from the lower limit to the upper limit of the 95% confidence intervals of the hazard ratios.
