# Supplementary material for: Egg-laying by female Aedes aegypti shapes the bacterial communities of breeding sites
Source: BMC Biol. 2023 Apr 26;21:97. doi: 10.1186/s12915-023-01605-2 (PMC10134544; doi:10.1186/s12915-023-01605-2)
Supplement: Supplementary file 8 — Additional file 8: Supplementary Table 1. Bacteria genera isolated from Aedes aegypti exposed to bacteria of the genera Asaia or Elizabethkingia and the control group. [file 12915_2023_1605_MOESM8_ESM.pdf]

Additional file 8

Supplementary table 1. Bacteria genera isolated from *Aedes aegypti* exposed to bacteria of the genera *Asaia* (+*Asaia*) or *Elizabethkingia* (+*Elizabethkingia*) and the control group.

Taxonomic identification	Source
Acinetobacter	Control
Bacillus	Control, +*Asaia*, +*Elizabethkingia*
Chryseobacterium	Control
Elizabethkingia	+*Elizabethkingia*
Enterobacter	Control, +*Elizabethkingia*
Exiguobacterium	+*Elizabethkingia*
Pseudomonas	+ *Asaia*
Stenotrophomonas	+ *Asaia*
